# Recombinant Mtb9.8 of *Mycobacterium bovis* stimulates TNF-α and IL-1β secretion by RAW264.7 macrophages through activation of NF-κB pathway via TLR2

**DOI:** 10.1038/s41598-018-20433-x

**Published:** 2018-01-31

**Authors:** Shuqing Liu, Hong Jia, Shaohua Hou, Ting Xin, Xiaoyu Guo, Gaimei Zhang, Xintao Gao, Ming Li, Wuyang Zhu, Hongfei Zhu

**Affiliations:** 1 Key Laboratory of Medical Virology, Ministry of Health, National Institute for Viral Disease Control and Prevention, Chinese Center for Disease Control and Prevention, Beijing, 102206 P. R. China; 2 Institute of Animal Sciences, Chinese Academy of Agricultural Sciences, Beijing, 100193 P. R. China

## Abstract

The Mtb9.8 antigenic protein of *Mycobacterium bovis/Mycobacterium tuberculosis* has been identified as a target of the T-cell response. However, the interaction of Mtb9.8 with Toll-like receptors (TLRs) and the relevant signaling pathways have not been fully clarified. In this study, recombinant Mtb9.8 (rMtb9.8) derived from *M. bovis*-stimulated RAW264.7 cells initiated the secretion of TNF-α and IL-1β in a dose-dependent manner. Blocking assays show that TLR2-neutralizing antibody decreases the production of TNF-α and IL-1β. Moreover, NF-κB activation is associated with TNF-α and IL-1β production by rMtb9.8 stimulation, and rMtb9.8 stimulation also induces the phosphorylation of NF-κB p65 at Ser536 and its rapid nuclear translocation in RAW264.7 cells. Furthermore, NF-κB luciferase activity is rapidly activated in response to rMtb9.8 in RAW264.7 cells and is also significantly increased in rMtb9.8-induced HEK293-TLR2. However, these activations were abrogated in cells with a dominant-negative mutation of NF-κB p65 and by treatment with anti-TLR2 antibody. We also find that rMtb9.8 induces the activation of IRF-1. These findings indicate that *M. bovis*-derived rMtb9.8 activates the NF-κB pathway via TLR2 in RAW264.7 cells. In particular, it phosphorylates NF-κB p65 at Ser536 and induces nuclear translocation, thereby leading to the production of TNF-α and IL-1β, which correlates with the induction of IRF-1.

## Introduction

*Mycobacterium bovis* is a member of the *Mycobacterium tuberculosis* complex (MTBC), which causes a significant zoonosis constituting a public health problem^1^. It is estimated that >50 million cattle worldwide are infected with *M. bovis*, costing three billion USD annually^2^. Host resistance to *M. tuberculosis* infection depends on both innate and adaptive immunity^3^. *M. tuberculosis* uses a repertoire of dampening signals to subvert the innate immune response^4^. During the early stages of *M. tuberculosis* infection, macrophages serve as the main effector cells that are involved in the activation of cytokine production in a complex process of cross-regulation that limits bacterial survival and proliferation^5^. This cytokine network plays a crucial role in the inflammatory response and in the outcome of mycobacterial infections^6^.

Tumor necrosis factor alpha (TNF-α) and interleukin-1 beta (IL-1β) are mainly produced by immune cells that are involved in the host response to *M. tuberculosis* and are overexpressed at sites involved in pulmonary tuberculosis, where they finally recruit macrophages and lymphocytes that seal up infectious foci by forming granulomas^7,8^. Toll-like receptors (TLRs) are the principal effectors of innate immunity and are essential for microbial recognition by macrophages and dendritic cells^9^. Several mycobacterial molecules, including ESAT-6^4^, lipomannan^10^, lipoarabinomannan^11^, 19-kDa lipoprotein^12^, LprG (Rv1411c)^13^, LprA^14^, MPT83^15^, TB10.4^16^ and 38-kDa lipoprotein^17^, activate immune cells that are dependent on TLR2. The binding of these pathogen-specific ligands to TLRs initiates signal transduction pathways in the host cell and culminates in the activation of nuclear factor-kappa B (NF-κB), the induction of cytokines and the elicitation of an adaptive immune response against the pathogen^18,19^. The NF-κB family of transcription factors plays an important role in the innate immune system and in adaptive immune responses. It consists of five members: p50, p52, p65 (RelA), c-Rel and RelB^20^. All NF-κB family members share an N-terminal Rel homology domain (RHD) that is responsible for dimerization, sequence-specific DNA binding and interaction with inhibitory IκB proteins^21^. NF-κB activation occurs in response to a variety of inflammatory agents; following activation, NF-κB translocates to the nucleus within minutes, resulting in the coordinated expression of multiple genes associated with inflammation and innate immunity^21^. Previous studies have indicated that NF-κB-dependent gene transcription can be regulated by bacillus Calmette–Guerin (BCG) mycobacteria and by several mycobacterial ligands for TLR2 in macrophages, ultimately leading to the production of diverse pro-inflammatory cytokines^11^.

The secretory proteins of *M. tuberculosis* have gained attention in recent years both as vaccine candidates and as diagnostic and therapeutic tools that target the immune system and trigger a putative protective response^22^. Mtb9.8, a recently identified secretory antigenic protein of the MTBC, is encoded by the EsxG (Rv0287) gene, which is also a member of the CFP-10/ESAT-6 family^23^. Moreover, Mtb9.8 can be recognized by peripheral blood mononuclear cells (PBMCs), in which it causes strong antigen-induced proliferation and/or IFN-γ secretion in TB patients but not in BCG-vaccinated healthy donors. Mtb9.8 has also been shown to stimulate the T-cell response in which Th1 cytokine secretion, including TNF-α and IL-12, is induced; because this response mediates protective immunity, Mtb9.8 provides protection as a new vaccine candidate for TB^24^. Previous reports indicated that Rv0287 and Rv0288 form a tight 1:1 complex, as observed for the CFP-10 and ESAT-6 complex^25^. We recently reported that *M. bovis*-derived rTB10.4 (Rv0288) induces the production of TNF-α, IL-6 and IL-12 p40 by RAW264.7 macrophages through activation of the MAPK and NF-κB pathways via TLR2^16^. We also demonstrated that rMtb9.8 of *M. bovis* triggers the production of IL-6 and IL-12 p40 by activation of the ERK, p38 and NF-κB pathways^26^. However, whether or not common TLR pathways are involved in this activation was not investigated, and little is known concerning the details of NF-κB translocation to the nucleus following rMtb9.8 activation. Since NF-κB is activated in infected cells and its activation has been reported to regulate the IL-1β-induced expression of interferon regulatory factor-1 (IRF-1)^27^, we asked whether IRF-1 is also regulated by rMtb9.8. The results of this study indicate that rMtb9.8 is able to induce the production of TNF-α and IL-1β in RAW264.7 cells and that it activates the NF-κB pathway through TLR2-mediated signaling, thereby contributing to the induction of IRF-1.

## Materials and Methods

Cell lines. The mouse macrophage cell line RAW264.7 and the human kidney cell line HEK293T were purchased from the American Type Culture Collection (Manassas, VA, USA) and cultured in Dulbecco’s Modified Eagle’s Medium (DMEM) (HyClone Laboratories; Logan, UT, USA). The human monocytic cell line THP-1(China Center for Type Culture Collection, Wuhan University, China) was cultured in RPMI 1640 (HyClone). All culture media contained 10% FBS (Life Technologies BRL; Gaithersburg, MD, USA), and the cultured cells were maintained at 37 °C in a humidified incubator (5% CO_2_). The HEK293-TLR2 stable cell line (HEK-TLR2) and the HEK293-TLR4 stable cell line (HEK-TLR4) were purchased from InvivoGen (San Diego, CA, USA).

### Recombinant expression of Mtb9.8 protein in *E. coli*

Rv0287 was cloned in pET30a (+) (Novagen, Madison, WI, USA) and expressed as a full-length protein with an N-terminal His tag in *E. coli* BL21 as reported previously^26^. The recombinant protein was purified by affinity chromatography using Ni-NTA (GE Healthcare, Germany) to test its ability to induce cytokine production in macrophages. The endotoxins were removed from rMtb9.8 using Triton X-114 two-phase separation as previously described^19,26^. All purified proteins were filtrated with a 0.22-μm sterile filter membrane. Protein concentrations were determined by bicinchoninic acid (BCA) assay.

### Cytokine ELISAs

Cytokine ELISA kits (R&D Systems; Wiesbaden-Nordenstadt, Germany) were used to measure cytokine levels in the culture supernatants of ligand-treated RAW264.7 cells. LPS (*E. coli* 0111:B4) was purchased from Sigma-Aldrich (St. Louis, MO, USA), and Pam_3_CSK_4_ was purchased from InvivoGen. In experiments designed to block TLR signaling, RAW264.7 cells were pre-treated for 30 min at 37 °C with 30 μg/ml of a mouse antibody (Ab) against TLR2, TLR4 or a mouse IgG isotype-matched control Ab (all from Imgenex). In experiments designed to block NF-κB signaling, RAW264.7 cells were pre-treated for 1 h at 37 °C with 1, 5 or 10 μM NF-κB-specific inhibitor (BAY 11-7082; Sigma-Aldrich) prior to rMtb9.8 exposure as described above. The cell culture supernatants were collected at 4 h, and the levels of TNF-α and IL-1β were measured using ELISA. The inhibitor was dissolved in 0.1% DMSO before addition to the cell culture, and DMSO alone was used as a negative control. rMtb9.8 (1 mg) was treated with proteinase K (50 μg/ml; Promega) at 37 °C for 24 h, heated at 98 °C for 10 min to inactivate the enzyme, and added at a concentration of 5 μg/ml to RAW264.7-seeded culture medium; the cells were cultivated in a 24-well culture plate as described above. The cell culture supernatant was used in the cytokine production assay as described above. In additional experiments, THP-1 cells were incubated with rMtb9.8 (5 µg/ml) for 4 h at 37 °C; the cell culture supernatants were then collected, and the level of TNF-α was measured using ELISA as described above.

### Transfection and luciferase reporter assays

Using FuGENE HD (Roche), RAW264.7 cells cultured in 24-well plates (Nunc, Roskilde, Denmark) were transiently co-transfected with various amounts of the indicated plasmids together with 0.2 μg/well of the reporter plasmid pNF-κB-Luc (Stratagene, Santa Clara, CA, USA) and 0.05 μg/well of the pRL-TK plasmid (Promega) for normalization. The expression plasmid pIRES-EGFP-pp65RHD (0.2 μg/well; a dominant-negative mutant of the NF-κB p65 plasmid; provided by Dr. Hegang Li) was overexpressed^28^ to inhibit the activation of NF-κB p65; 0.2 μg/well of the expression plasmid pIRES2-EGFP was used as the empty control. The total amount of transfected DNA was kept constant by the addition of the empty vector. After 24 h, the transfected cells were stimulated with rMtb9.8. In experiments designed to evaluate NF-κB activation by TLR2 or TLR4, RAW264.7 cells were pre-treated for 30 min at 37 °C with 30 μg/ml of a mouse Ab against TLR2, TLR4 or a mouse IgG isotype-matched control Ab (isotype Ab) (all from Imgenex). pNF-κB-Luc expression after rMtb9.8 stimulation was measured using a luciferase assay after transfection with the reporter plasmid, pNF-κB-Luc (0.2 μg/well). The pRL-TK plasmid (0.05 μg/well) was used for normalization. pNF-κB-Luc expression after rMtb9.8 stimulation of a HEK293-TLR2 stable cell line, a HEK293-TLR4 stable cell line, and a HEK293T cell line was assayed using the NF-κB dual-luciferase reporter assay system (Promega) according to the manufacturer’s instructions. Cell extracts were collected at the indicated time points after incubation and assayed for luciferase activity. Firefly and Renilla luciferase activities were monitored in a MicroBeta TriLux liquid scintillation counter and a luminometer (Turner BioSystems, Sunnyvale, CA, USA), respectively, according to the manufacturers’ instructions. Normalized reporter activity was expressed as the mean firefly luciferase value divided by the mean Renilla luciferase value obtained in three independent and representative experiments. All reporter assays were repeated a minimum of three times.

### ImageStream analysis of NF-κB translocation

ImageStream is a high-throughput imaging flow cytometry system that performs multidimensional pixel analysis of individual cells passing through a flow cell; it is a statistically robust method for measuring the relative amounts of cytoplasmic and nuclear components^29^. RAW264.7 cells were stimulated with rMtb9.8 (5 μg/ml) for 10 min or 30 min at 37 °C. After stimulation, the cells were fixed and incubated overnight in 100 μl permeabilization/wash buffer (0.1% TritonX-100/2% FBS/0.1% sodium azide/PBS) containing a rabbit antibody against phospho-NF-κB (#3033; Cell Signaling, Beverly, MA, USA) at 1:20 dilution (10 μg/ml) at 4 °C. The cells were then washed once and incubated for 1 h in 100 μl permeabilization/wash buffer containing an AF488-conjugated anti-rabbit IgG secondary antibody (#4412; Cell Signaling) at 25 °C. After incubation, the cells were washed once and resuspended in 50 μl of wash buffer containing DAPI (#4083; Cell Signaling) at 0.1 μg/ml. Fluorescent images were obtained (50,000 events per condition) using an Amnis ImageStream100. A mask was created on the nucleus, and the colocalization of TFs and nuclear dye was measured by similarity within the area (IDEAS software; Amnis, Seattle, WA, USA)^29^.

### Protein preparation and Western blot analysis

RAW264.7 cells were stimulated with rMtb9.8 (5 μg/ml) for various amounts of time, lysed and purified by centrifugation using the M-PER Mammalian Protein Kit or the NE-PER Nuclear and Cytoplasmic Extraction Kit (Pierce, Rockford, IL, USA). The cell lysis buffer was supplemented with a proteinase inhibitor (Pierce) and a phosphatase inhibitor (Roche, Indianapolis, IN, USA). Extracted total and nuclear proteins were separated by SDS-PAGE and electrophoretically transferred to polyvinylidene difluoride membranes (Millipore Corp., MA, USA). After blocking with 5% nonfat milk in TBST (20 mM Tris-HCl, 15 mM NaCl, 0.05% (v/v) Tween-20, [pH 7.4]), the membranes were incubated with primary antibodies overnight at 4 °C. The antibodies used were rabbit anti-mouse IRF-1, rabbit anti-mouse TATA-binding protein (TBP), and rabbit anti-mouse β-actin (both from Cell Signaling Technology, Beverly, MA, USA). The membranes were washed with TBS and incubated for 1 h at 25 °C with the appropriate HRP-conjugated anti-rabbit IgG secondary Ab (1:5000) (Pierce). The peroxidase-positive bands were detected using SuperSignal West Dura Extended Duration Substrate (Pierce) and visualized by measuring their chemical luminescence (LAS-4000; FUJIFILM, Japan).

### Statistical analysis

The results are presented as the mean ± SD of triplicate experiments. Statistical analysis was performed using one-way ANOVA followed by Tukey’s test using SigmaStat 3.5 software (Systat Software, Richmond, VA, USA). Values of *p < 0.05, **p < 0.01, and ***p < 0.001 were considered statistically significant.

## Results

### rMtb9.8 stimulates the production of TNF-α and IL-1β by RAW264.7 mouse macrophages

**Figure 1:**
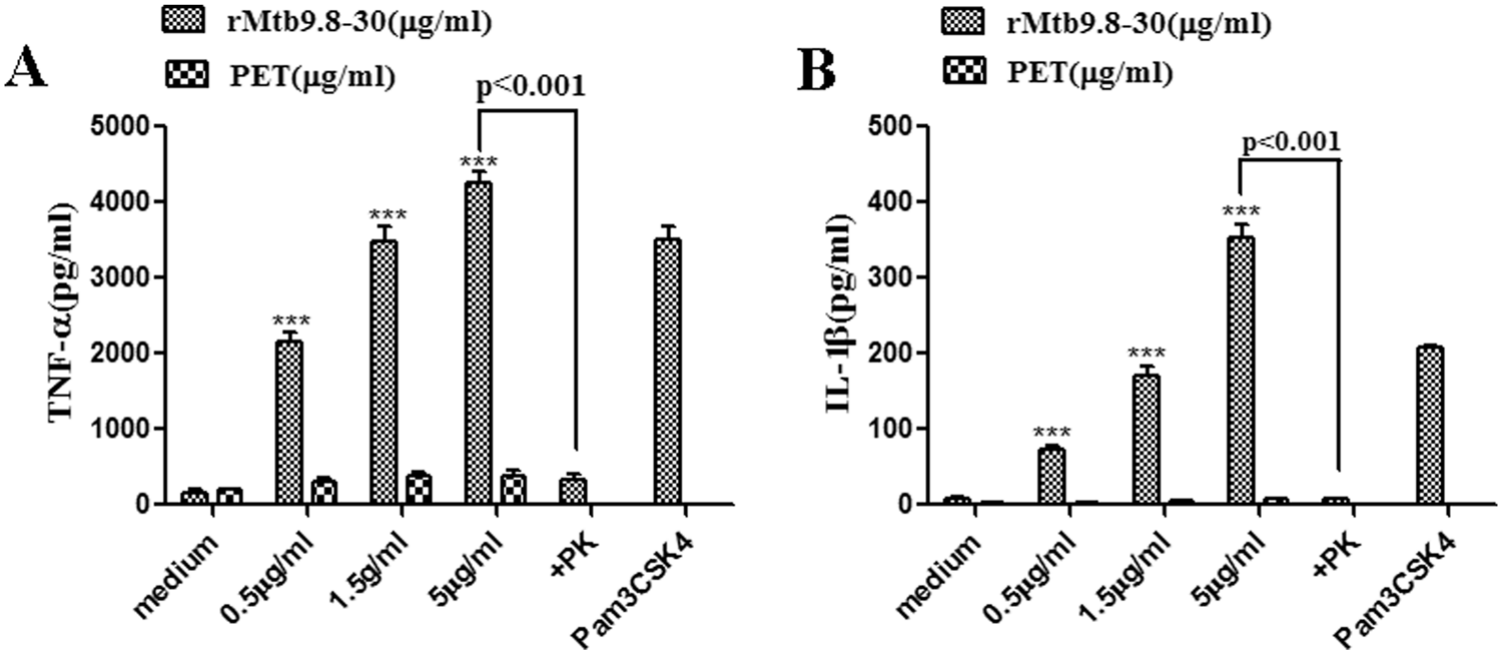
rMtb9.8 induces the secretion of TNF-α (**A**) and IL-1β (**B**) by mouse macrophages. RAW264.7 cells were cultured in the presence of various concentrations of rMtb9.8 (0, 0.5, 1.5, or 5 µg/ml). Pam3CSK4 (10 µg/ml), a TLR1/2 agonist, was used as a positive control. The pET30 vector tag protein without the Mtb9.8 gene (PET) was used as a negative control. Proteinase K abrogated the rMtb9.8-induced expression of TNF-α and IL-1β (p < 0.001). After 4 h of incubation, the cell culture supernatants were collected, and the levels of TNF-α (**A**) and IL-1β (**B**) were measured by ELISA. The data are expressed as the mean ± SD of the results of three separate experiments. **p < 0.01, ***p < 0.001, stimulated cells versus cells cultured in medium alone.

**Figure 2:**
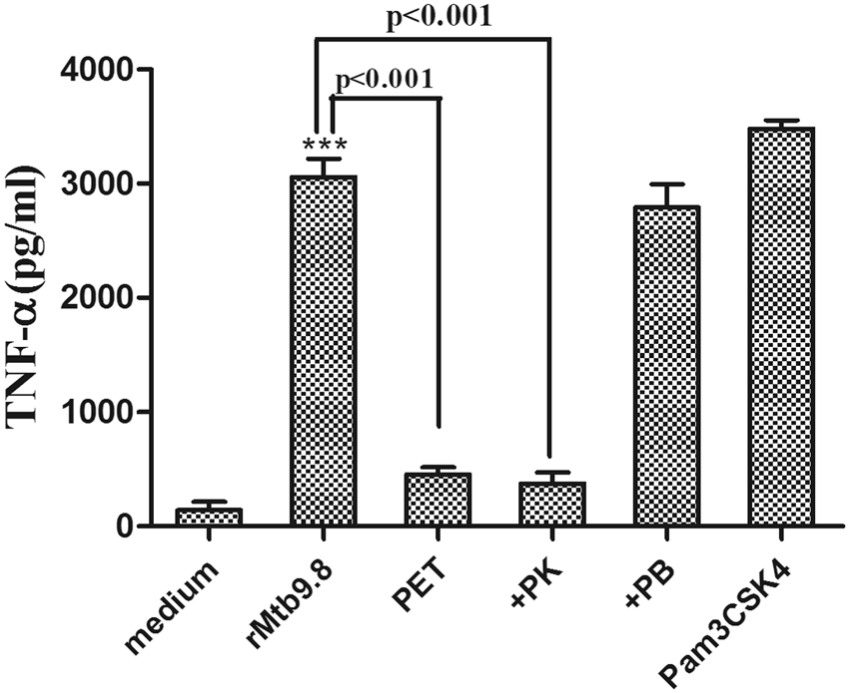
rMtb9.8 induces the secretion of TNF-α by THP-1 cells. THP-1 cells were cultured in the presence of rMtb9.8 (5 µg/ml) for 4 h at 37 °C. The cell culture supernatant was collected, and the level of TNF-α in the supernatant was measured using ELISA. Pam3CSK4 (10 µg/ml), a TLR1/2 agonist, was used as a positive control. The pET30 vector tag protein without the Mtb9.8 gene (PET) was used as a negative control. Proteinase K abrogated rMtb9.8-induced expression of TNF-α (p < 0.001), as described above. rMtb9.8 contaminated by LPS may contribute to the expression of TNF-α; therefore, polymyxin B (PB), an inhibitor of LPS, was used.

We recently demonstrated the ability of rMtb9.8 to stimulate the production of IL-6 and IL-12 p40 by RAW264.7 cells through the MAPK and NF-κB pathways^26^. We, therefore, sought to determine whether rMtb9.8 could activate the production of TNF-α and IL-1β. Cytokine levels in the cell culture supernatants were measured by ELISA after incubation of the cells with rMtb9.8 (0.5, 1.5, or 5 µg/ml) or Pam3CSK4 (10 μg/ml) for 4 h. To exclude the possibility that *E. coli*-derived contaminants were present in the recombinant preparation, a pET30 vector lacking the Mtb9.8 gene was used as a negative control in *E. coli*. As shown in Figs 1A and B, exposure of RAW264.7 cells to rMtb9.8 resulted in the production of significantly higher levels of TNF-α and IL-1β in a dose-dependent manner compared with the negative control (cells to which PET tag protein or medium was added) (p < 0.001). The PET tag protein or medium solution did not affect TNF-α or IL-1β production. Proteinase K abrogated the rMtb9.8-induced increase in the expression of TNF-α and IL-1β (p < 0.001). Although endotoxin contamination of prepared rMtb9.8 was removed by exposure of the material to Triton X-114, we also investigated whether its pro-inflammatory activity was due to LPS contamination by measuring the sensitivity of the rMtb9.8 effect to polymyxin B (PB). rMtb9.8 was pre-treated with 10 µg/ml PB to inhibit any possible LPS-induced increase in TNF-α and IL-1β production. PB treatment did not affect the rMtb9.8-induced increase in the secretion of TNF-α and IL-1β (data not shown). The results suggest that the production of TNF-α and IL-1β is stimulated by rMtb9.8 in RAW264.7 cells and that the observed effect is not due to LPS contamination. To formally rule out the possibility that other contributing factors might be responsible for the observed effects, we evaluated the capacity of rMtb9.8 to enhance the production of TNF-α by THP-1 cells (Fig. 2). We found that the release of TNF-α through activating THP-1 cells also could be induced by rMtb9.8 (5 µg/ml), which can be abrogated by Proteinase K (p < 0.001). Pretreatment with polymyxin B or PET tag protein had no effect on TNF-α production. Because rMtb9.8 elicited similar effects in RAW264.7 cells and THP-1 cells, all subsequent experiments reported here were performed in RAW264.7 cells.

### rMtb9.8 stimulation of the production of TNF-α and IL-1β by RAW264.7 mouse macrophages requires TLR2

**Figure 3:**
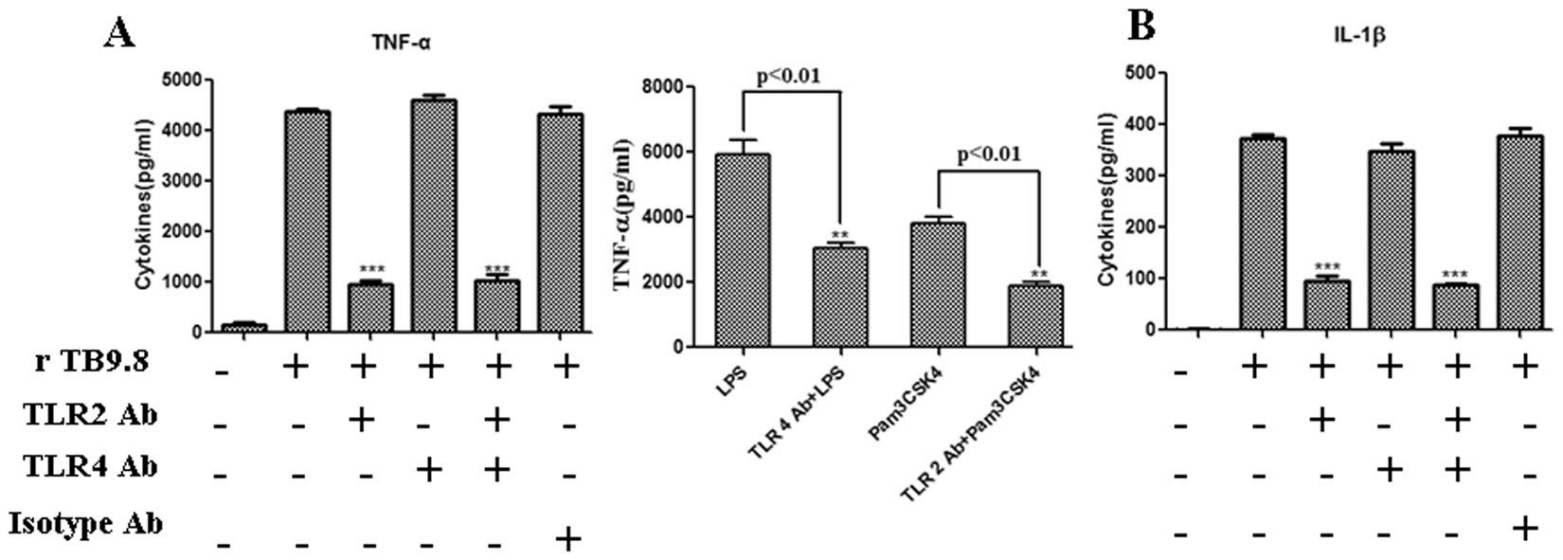
TLR2 is required for the secretion of TNF-α and IL-1β by rMtb9.8-induced mouse macrophages. RAW264.7 cells were pre-treated for 30 min at 37 °C with anti-TLR2 (30 µg/ml), anti-TLR4 (30 µg/ml) or isotype Ab (30 µg/ml) before stimulation with rMtb9.8 (5 μg/ml) for 30 min. LPS and Pam3CSK4 were used as positive controls to demonstrate the blocking effects of TLR4 and TLR2 antibodies. The levels of TNF-α (**A**) and IL-1β (**B**) in the supernatant were measured by ELISA. The data are expressed as the mean ± SD of the results of three separate experiments. **P < 0.01, ***P < 0.001, stimulated cells versus cells cultured in medium alone.

In *M. tuberculosis* infection, TLR2 and TLR4 have been shown to mediate the cellular responses to diverse mycobacteria. TLR2 results in the production of pro-inflammatory molecules that contribute to both host protection and immunopathology^15^. We investigated whether TLR2 and TLR4 are involved in rMtb9.8 activation. The production of TNF-α and IL-1β by RAW264.7 cells after rMtb9.8 induction was measured after pre-incubation of the cells with blocking antibody to TLR2 or TLR4 or with an isotype Ab for 30 min. LPS and Pam3CSK4 were used as positive controls to demonstrate the blocking effects of the TLR4 and TLR2 antibodies. As shown in Fig. 3A,B, blockade of TLR2 signals significantly decreased the production of TNF-α (p < 0.001; ~75% inhibition at 30 μg/ml) and IL-1β (p < 0.001; ~55% inhibition at 30 μg/ml) after rMtb9.8 stimulation. However, anti-TLR4 and isotype Abs did not have these effects in RAW264.7 cells. These results indicate that the rMtb9.8-induced production of TNF-α and IL-1β is mediated by TLR2.

**Figure 4:**
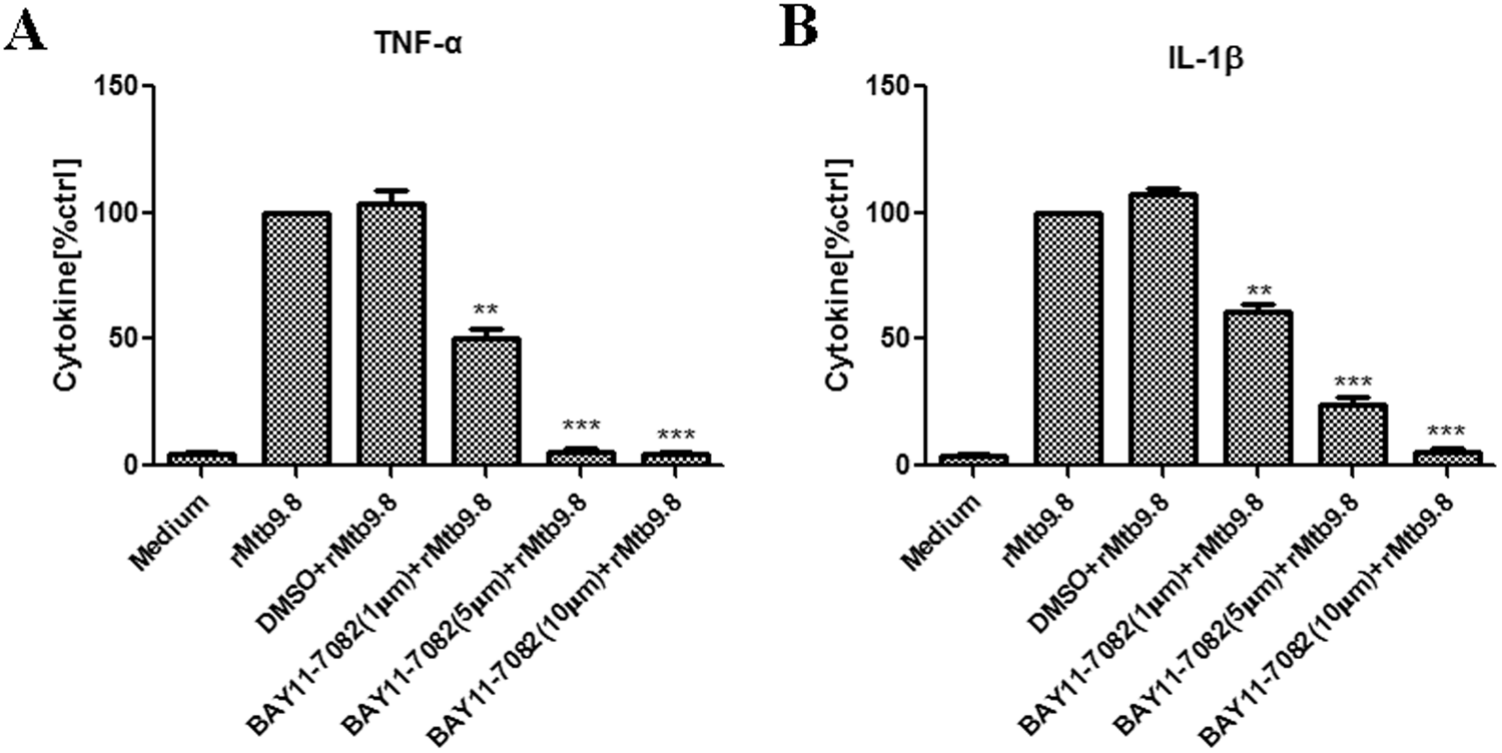
NF-κB is required for the secretion of TNF-α and IL-1β by rMtb9.8-induced mouse macrophages. RAW264.7 cells were pre-treated with the specific NF-κB inhibitor BAY 11-7082 at concentrations of 1, 5 or 10 μM for 1 h. Four hours after stimulation with rMtb9.8 at 5 µg/ml, the culture supernatants were collected and the levels of TNF-α (**A**) and IL-1β (**B**) were measured. The level of rMtb9.8-induced TNF-α or IL-1β was set to 100, and the relative decrease in cytokine production in the presence of each inhibitor was calculated and expressed as the percentage of control (% ctrl). The data are expressed as the mean ±SD of the results of three independent experiments. **P < 0.01, ***P < 0.001, cells treated with rMtb9.8 and the inhibitor versus cells stimulated with rMtb9.8 alone.

**Figure 5:**
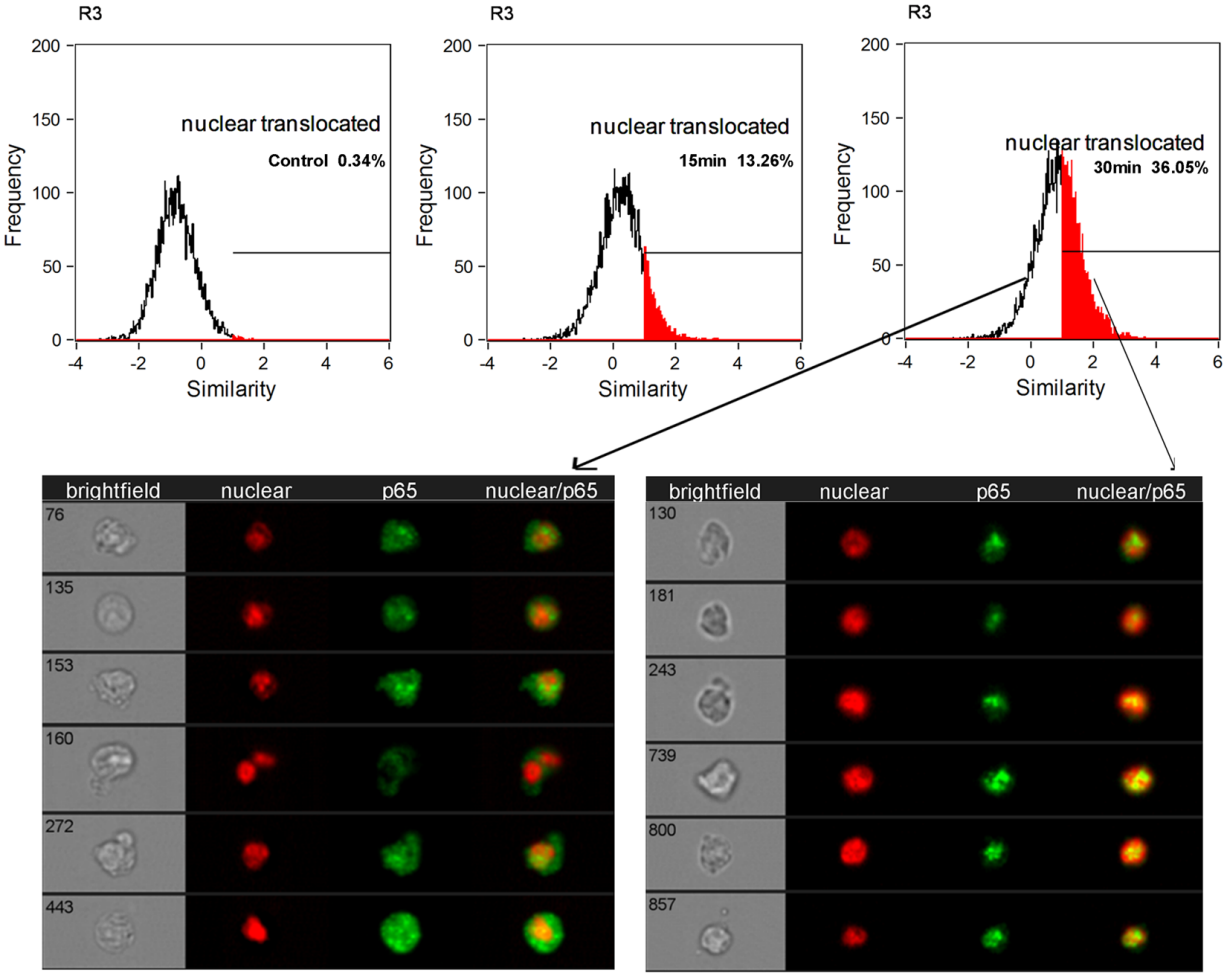
rMtb9.8 induces the phosphorylation of NF-κB p65 at Ser536 and nuclear translocation in mouse macrophages. Analysis of the nuclear translocation of NF-κB in RAW264.7 cells incubated with rMtb9.8 (5 µg/ml) for 0, 10 and 30 min by ImageStream 100 is shown. A specific antibody against phospho-NF-κB was used to detect NF-κB phosphorylated at Ser536. Live cells were distinguished from dead cells by the presence of a single, well-formed DAPI-stained nucleus (live cells), after which we gated live cells and NF-κB-containing cells. Nuclear translocation was defined by the colocalization of NF-κB (green) with the nuclear stain DAPI (red); colocalization is indicated by the presence of a bright yellow nucleus in the overlaid images of live cells and focused NF-κB-containing cells. Top: From left to right, the images show cells that exhibit low to high nuclear translocation at time intervals of 0, 15 and 30 min. Bottom: From left to right, the images are as follows: bright field, DAPI stained, phospho-p65-stained and a merged image of DAPI and phospho-p65-stained cells. The data are representative of three independent experiments; 30,000–50,000 live focused cells were recorded in each experiment.

**Figure 6:**
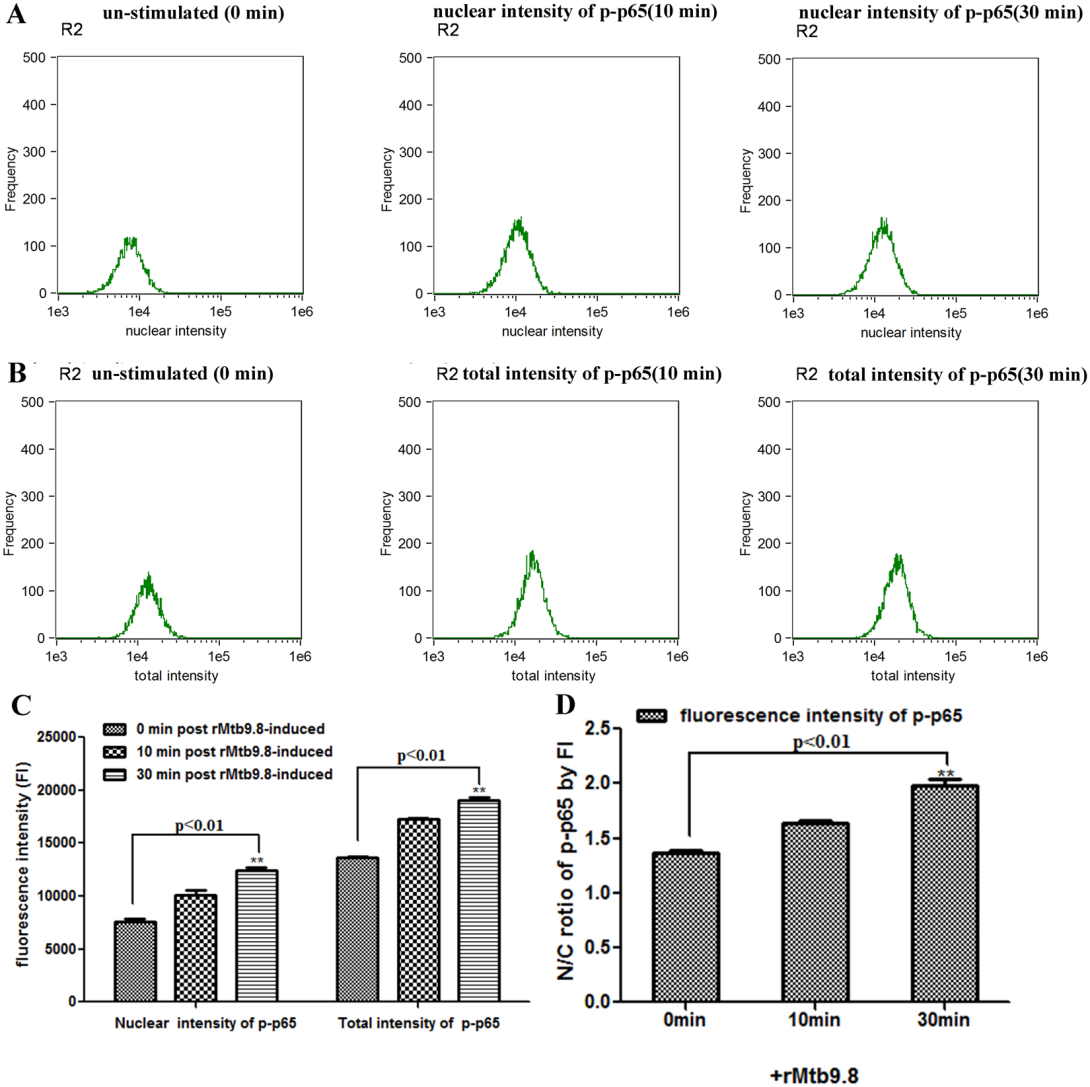
rMtb9.8 increases the nuclear and total amounts of phosphorylated NF-κB p65. RAW264.7 cells were cultured in the presence of rMtb9.8 (5 µg/ml) for the indicated times (0, 10, 30 min), after which a total of 1 × 10^4^ cells were stained as described in the Materials and Methods section; images of the cells were acquired using the ImageStream 100. The nuclear fluorescence intensity and total fluorescence intensity of phosphorylated p65 were analyzed using IDEAS 6.0 software. The cells were stained for phosphorylated p65 and with the nuclear dye DAPI following rMtb9.8 activation; the histograms show the nuclear fluorescence intensity (**A**) and the total fluorescence intensity of phosphorylated p65 (**B**) of cells incubated with rMtb9.8 for the indicated times. (**C**) The nuclear fluorescence intensity and total fluorescence intensity of phosphorylated p65 following rMtb9.8 activation for 0, 10 and 30 min were analyzed by fluorescence intensity (FI) using IDEAS 6.0 software. The results show that the nuclear fluorescence intensity of phosphorylated p65 and the total fluorescence intensity of phosphorylated p65 increased significantly at 30 min after rMtb9.8 induction compared with that in unstimulated cells. (**D**) The nuclear-to-cytoplasmic ratio (N/C ratio) of phosphorylated p65 following rMtb9.8 activation was analyzed by fluorescence intensity (FI) using IDEAS 6.0 software. The results show that the mean and SEM of the N/C ratio of phosphorylated p65 dramatically increased by 30 min following rMtb9.8 activation as measured by fluorescence intensity (FI). The data shown represent one of three independent experiments. **P < 0.01, ***P < 0.001, stimulated cells versus cells cultured in medium alone.

rMtb9.8 stimulates the production of TNF-α and IL-1β by RAW264.7 mouse macrophages through the phosphorylation of transcriptional factor NF-κB and induces the activation of IRF-1 NF-κB can be activated within minutes by a variety of stimuli, including inflammatory cytokines such as TNF-α and IL-1β^30^. Thus, we investigated whether NF-κB plays a role in the induction of TNF-α and IL-1β by rMtb9.8 stimulation. RAW264.7 cells were pre-treated with the NF-κB-specific inhibitor BAY 11-7082 at 1, 5 or 10 µM for 1 h prior to rMtb9.8 stimulation. As shown in Fig. 4A,B, cells treated with BAY 11-7082 showed concentration-dependent inhibition of TNF-α and IL-1β expression after rMtb9.8 induction, whereas DMSO alone had no inhibitory effect. We next measured the viability of the cells after exposure to increasing concentrations of BAY 11-7082 using a real-time cell electronic sensing (RT-CES) system. Treatment of RAW264.7 cells with BAY 11-7082 at concentrations of 1, 5 or 10 μM for 24 h did not result in the detection of any cellular toxicity by the RT-CES system, excluding nonspecific cytotoxicity as a possible explanation for the decreased cytokine output (data not shown). These observations suggest that rMtb9.8 induces the production of TNF-α and IL-1β in RAW264.7 cells specifically through the NF-κB signaling pathway. Potent NF-κB activators such as TNF-α and IL-1β cause almost complete degradation of IκBs (especially IκBα), leading to NF-κB phosphorylation and translocation to the nucleus within minutes^31,32^. Thus, we next sought to quantify the degree of NF-κB p65 phosphorylation at the Ser536 site and to measure the nuclear translocation of NF-κB. Using an antibody to phospho-NF-κB and the ImageStream 100 system, we collected 30,000 to 50,000 live focused cells and fluorescent cell images. To eliminate the incidence of false positives, nuclear translocation was defined as the colocalization of NF-κB p65 with material stained by the fluorescent DNA dye DAPI (Fig. 5). rMtb9.8 stimulated the nuclear translocation of NF-κB p65 5 min after treatment in a time-dependent manner, reaching 13.26% at 15 min. The percentage of phosphorylated p65 in the nucleus peaked at 36.05% by 30 min. These levels decreased 1 h after rMtb9.8 stimulation (data not shown). In unstimulated cells, NF-κB p65 nuclear translocation was extremely low (0.34%). We further analyzed the nuclear fluorescence intensity (FI) and total FI of anti-phospho-p65 using IDEAS 6.0 software (Fig. 6). In comparison with unstimulated cells, the nuclear fluorescence intensity of phosphorylated p65 and the total fluorescence intensity of phosphorylated p65 at 30 min increased significantly following exposure to rMtb9.8. As the amount of rMtb9.8 was increased, the nuclear-to-cytoplasmic ratio (N/C ratio) of phosphorylated p65 measured by FI increased in a time-dependent manner, and a significant increase occurred by 30 min compared to the ratio in unstimulated cells. These results suggest that cytoplasmic NF-κB p65 phosphorylated at the Ser536 site translocates to the nucleus following exposure of the cells to rMtb9.8. Taken together, the data suggest that rMtb9.8 induces the phosphorylation and nuclear translocation of NF-κB p65 following the production of TNF-α and IL-1β.

**Figure 7:**
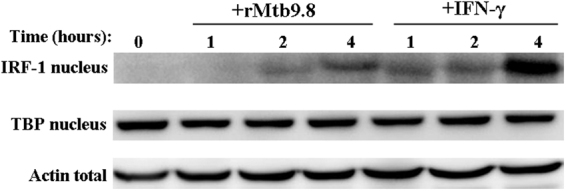
rTB9.8 induces the nuclear translocation of IRF-1. RAW264.7 cells were cultured in the presence of rTB9.8 (5 µg/ml) for the indicated times, and cell lysates of total and nuclear proteins were prepared. Western blot analysis was used to examine the nuclear translocation of IRF-1. The cells were also stimulated with IFN-γ (10 U/ml), a known potent inducer of IRF-1 expression, as a positive control. Bottom, reprobing with anti-TBP and anti-β-actin to ensure equal loading. No IRF-1 protein was detectable in unstimulated cells at any of the time points examined. The data shown represent one of three independent experiments. The full-length blots are presented in Supplementary Figure 1.

Key elements of the innate and adaptive immune response to *M. tuberculosis* that involve IRF-1 include^33^. The interaction of mycobacterial constituents with TLRs leads to the induction of IRF-1 through the activation of NF-κB and the expression of cytokines such as IL-1β, TNF-α, IFN-α,/β, IFN-γ, IL-12 and iNOS in the infected cells^33–35^. Taking that into consideration, we investigated the effects of rMtb9.8 on the activation of IRF-1 proteins. RAW264.7 cells were stimulated with rMtb9.8 (5 µg/ml) for various amounts of time and immunoblotted for IRF-1. As shown in Fig. 7, rMtb9.8-induced nuclear translocation of IRF-1 occurred within 2 h after exposure to rMtb9.8, and nuclear translocation was sustained at 4 h. In cells stimulated with IFN-γ (10 U/ml) as a positive control, IRF-1 protein expression was observed as early as 1 h, and the level increased at 4 h. In contrast, no IRF-1 protein was detectable in unstimulated cells at any of the time points examined. Taken together, these results show that, in addition to stimulation of the phosphorylation of NF-κB p65, rMtb9.8 also causes the activation of IRF-1.

### rMtb9.8-induced activation of the NF-κB pathway is regulated by TLR2 signaling

**Figure 8:**
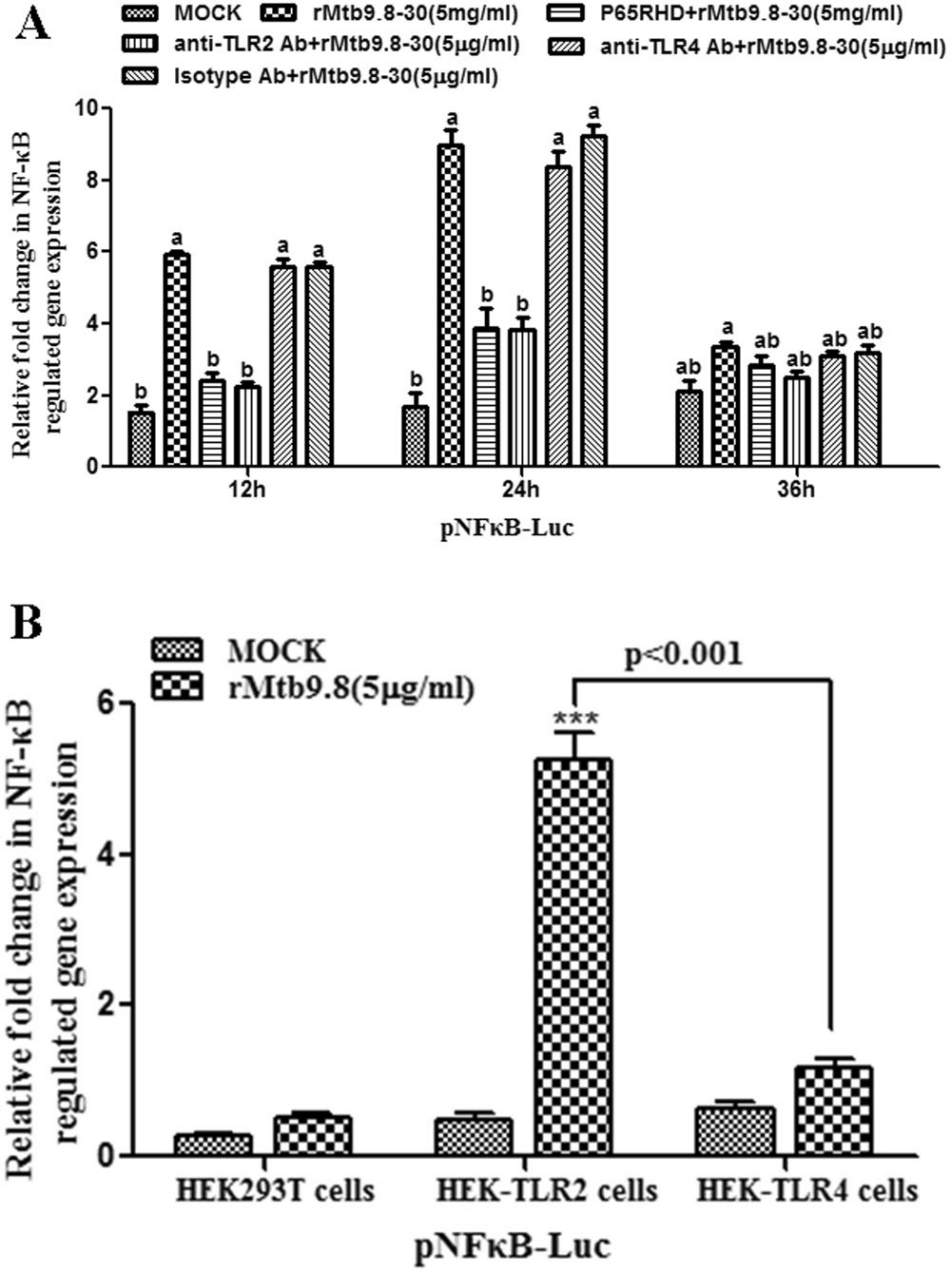
TLR2 is required for NF-κB activation by rMtb9.8. (**A**) RAW264.7 cells were transfected with the dual luciferase assay reporter vector pNF-κB-luc and the Renilla luciferase reporter vector pRL-TK. Exposure of the cells to rMtb9.8 significantly induced luciferase activity (P < 0.01) in a time-dependent manner, and this increase was significantly attenuated (P < 0.01) in cells transfected with the dominant-negative mutant of the NF-κB p65 plasmid pp65RHD. RAW264.7 cells were pre-treated with a mouse Ab against TLR2 or TLR4 or with an isotype Ab, and the luciferase activity due to pNF-κB-Luc expression was measured after rMtb9.8 stimulation. The Ab against TLR2 significantly decreased the activation of NF-κB luciferase activity after rMtb9.8 treatment, but the antibody against TLR4 and the isotype Ab did not. The data shown are representative of at least three independent experiments and are reported as the mean ± SD (n = 3). Different letters (a,b) indicate significant differences between groups (P < 0.01). (**B**) HEK293T cells, HEK-TLR2 cells and HEK-TLR4 cells were transfected, and the dual luciferase assay was performed. Luciferase activity was significantly induced by rMtb9.8 in HEK-TLR2 cells compared with HEK-TLR4 cells or untreated cells (P < 0.001). The data shown are representative of at least three independent experiments and are reported as the mean ± SD (n = 3).

The binding of ligands to TLRs, which triggers signal transduction that results in pro-inflammatory responses, is mediated by the activation of NF-κB^36^. Thus, we sought to determine whether TLR2 contributes to the rMtb9.8-induced activation of NF-κB using an NF-κB dual-luciferase reporter assay system. After rMtb9.8 stimulation of RAW264.7 cells, NF-κB luciferase activity was significantly increased (p < 0.01), and it peaked at 24 h (Fig. 8A). A plasmid carrying a dominant-negative mutant of NF-κB p65 (pIRES-EGFP-p65RHD) and pNF-κB-Luc were co-transfected into RAW264.7 cells. Overexpression of pIRES-EGFP-p65RHD significantly inhibited luciferase activity after rMtb9.8 activation (p < 0.01), but co-transfection of the cells with pNF-κB-Luc and the empty vector pIRES2-EGFP had no inhibitory effect. This result suggests that the observed NF-κB luciferase activity was stimulated by rMtb9.8. We next investigated whether the rMtb9.8-induced activation of NF-κB p65 is affected by TLR2. RAW264.7 cells were pre-treated with a mouse Ab against TLR2 or TLR4 or with an Ab of the same isotype, and the luciferase activity indicating pNF-κB-Luc expression was measured after rMtb9.8 stimulation using the NF-κB dual-luciferase reporter assay system. As shown in Fig. 8A, blockade of TLR2 signals significantly decreased NF-κB luciferase activity after rMtb9.8 stimulation. However, anti-TLR4 or isotype Ab did not have the same effect. This result indicates that the induction of NF-κB activation by rMtb9.8 is mediated by TLR2. This result was confirmed using the HEK293-TLR2 stable cell line (HEK293-TLR2), the HEK293-TLR4 stable cell line (HEK293-TLR4) and the HEK293T cell line, which were transfected with pNF-κB-Luc and expressed NF-κB luciferase activity after rMtb9.8 stimulation as above. As shown in Fig. 8B, treatment of HEK293-TLR2 cells with rMtb9.8 significantly increased the activation of NF-κB luciferase activity compared with that in HEK293-TLR4 cells or unstimulated cells (p < 0.001), whereas the NF-κB luciferase activity of HEK293-TLR4 cells was low despite exposure of the cells to rMtb9.8. HEK293T cells do not express TLR2; therefore, NF-κB luciferase activity was low even after rMtb9.8 stimulation. These observations suggest that the NF-κB activation induced by rMtb9.8 stimulation is dependent on TLR2.

## Discussion

MTBC are intracellular bacteria that represent a major global health challenge^1^, and a coordinated response of the innate and adaptive immune systems is required to control infection^6^. The pro-inflammatory cytokines involved in the host response to *M. tuberculosis*, which include TNF-α and IL-1β, are mainly produced by monocytes, macrophages and dendritic cells^8^. In patients with tuberculosis, IL-1β is expressed in excess and at the site of disease^37^. IL-1β production is significantly increased in tuberculosis pleurisy, which usually presents as a self-resolving type of primary tuberculosis^38^. Secretion of TNF-α by *M. tuberculosis-*infected macrophages is a potent mechanism for inducing the release of IFN-γ and killing *M. tuberculosis*. The ability of TNF-α to induce macrophage apoptosis is also important in the cross-presentation of *M. tuberculosis* antigens for CD8 + cytotoxic T cell priming, and it thereby indirectly contributes to the reduction of bacterial burden^39,40^. We chose RAW mouse macrophage cell lines as a research model for this study because they demonstrate a high level of TLR expression and possess a typical signal transduction system involving well-characterized signaling pathways in response to extracellular stimuli. We previously reported that rMtb9.8 induced the production of IL-6 and IL-12 by RAW264.7 cells and that the secretion of TNF-α could be activated in rTB10.4-induced macrophages^19,36^. In this study, we showed that stimulation of RAW264.7 cells by rMtb9.8 also significantly increased the production of the pro-inflammatory cytokines TNF-α and IL-1β. The production of cytokines did not occur when rMtb9.8 was digested with proteinase K, similar to the effect observed with other mycobacterial proteins such as 19-kDa mycobacterial lipoprotein^12^, rMPT83^15^, rTB10.4^16^ and 38-kDa Ag^17^. These data indicate that rMtb9.8 induces the production of pro-inflammatory cytokines by RAW264.7 cells and suggest that rMtb9.8 stimulation may enhance the innate immune response and accelerate Th1 cell activation.

The activation and nuclear translocation of classical NF-κB dimers (mainly p50–RelA) is associated with the increased transcription of genes encoding chemokines, cytokines and adhesion molecules^20,21^. In fact, NF-κB binding sites have been identified in the promoter regions of the IL-1β converting enzyme protease, c-myc and TNF-α genes^20^. There are also indirect pathways that lead to NF-κB activation, as illustrated by infection of pulmonary epithelial cells with *M. tuberculosis*, which results in the release of IL-1 and activation of the classical NF-κB pathway in monocytes^7^. Our previous report demonstrated that rMtb9.8 of *M. bovis* activates the ERK, p38 and NF-κB pathway and triggers the production of IL-6 and IL-12 p40. In this regard, we found that rMtb9.8-mediated induction of TNF-α and IL-1β in RAW264.7 cells was markedly attenuated by the NF-κB inhibitor BAY 11-7082 in a dose-dependent manner. These data support the idea that the effects of rMtb9.8 on cytokine production are mediated by the NF-κB signaling pathway. Potent NF-κB activators such as IL-1 and TNF-α cause almost complete degradation of IκBs (especially IκBα) within minutes, leading to the phosphorylation of Rel A/p65 and the subsequent activation and nuclear translocation of NF-κB^31,32^. Rel A/p65 is also subject to phosphorylation at two sites within the C-terminal TAD region (Ser529 and Ser536)^41^. After TNF-α or IL-1β treatment, Ser536 is phosphorylated by IKKs^42,43^. IKK β has been implicated in the direct phosphorylation of NF-κB p65 at Ser536 *in vitro* and vivo^41,44^. Thus, we used an antibody to phospho-NF-κB to specifically detect NF-κB phosphorylated at Ser536 and to quantify the nuclear translocation of NF-κB p65 following rMtb9.8 stimulation. As shown by ImageStream analysis, NF-κB p65 phosphorylation at Ser536 and translocation of NF-κB p65 to the nucleus in RAW264.7 cells increased following rMtb9.8 stimulation, leading to the downstream expression of TNF-α and IL-1β. Whether other phosphorylation sites are also involved in the NF-κB translocation to the nucleus by rMtb9.8 activation is unclear and remains to be further studied.

Interferon regulatory factor-1 (IRF-1) has been shown to be necessary for the host defense against infection by *M.tuberculosis* in mice^45^. Direct or autocrine induction of IRF-1 in infected cells by activation of NF-κB or the Jak-Stat pathway or both would be part of innate immunity. In addition, NF-κB has been shown to activate the expression of IRF-1 in an IL-1β-dependent manner. TNF-α, which regulates IRF-1 expression, as does activation of NF-κB in response, is also critical for the host response to *M. tuberculosis*^46^. Our data show that the presence of IRF-1 in the nucleus was induced by rMtb9.8, resulting in IRF-1 activation. However, whether or not activation of NF-κB by rMtb9.8 is involved in the nuclear translocation of IRF-1 is less clear and remains to be further studied.

Many *M. tuberculosis* protein and non-protein antigens modulate TLR signaling and thereby play roles in immune evasion^47^. Numerous studies have implicated TLR2 in immune recognition and in the responses of immune cells such as macrophages to stimulation by a variety of *M. tuberculosis* antigens; such stimulation leads to the initiation of pro-inflammatory cytokines in macrophages and dendritic cells through the activation of the NF-κB and MAPK pathways^15,16^. Interestingly, Mycobacterium avium infection and pro-inflammatory cytokines increase the cell surface expression of TLR2^48^. In RAW264.7 cells, TNF-α plus IFN-γ induced NO production and reduced the viability of intracellular *M. tuberculosis* due to the effect of the 19-kDa lipoprotein^12^. Phosphatidyl inositol dimannoside (PIM2) and hexamannoside (PIM6) in BCG and H37Rv were recently shown to stimulate macrophages to secrete TNF-α through TLR2^11^. Thus, we investigated whether rMtb9.8 induces the expression of TNF-α and IL-1β via the TLR2-mediated pathway in RAW264.7 cells. The rMtb9.8-induced production of TNF-α and IL-1β was found to be significantly inhibited by the blocking effect of anti-TLR2, but this was not observed in anti-TLR4 or untreated cells. These results demonstrate that TLR2 is necessary for the signaling associated with rMtb9.8 activation, consistent with previous results showing that the recombinant mycobacterial proteins ESAT-6^4^, LprG (Rv1411c)^13^, LprA^14^, MPT83^15^, TB10.4^16^ and 38-kDa lipoprotein^17^ trigger TLR2 expression that results in the secretion of cytokines such as TNF-α. In addition, our results showed that NF-κB luciferase activity was activated rapidly and specifically in response to rMtb9.8 and that this effect was abrogated in a dominant-negative mutant of NF-κB p65 RHD and significantly decreased by the blockade of TLR2 signals. Moreover, rMtb9.8 activated NF-κB luciferase more strongly in the HEK293-TLR2 cell line than in HEK293-TLR4 cells or unstimulated cells, although the absence of TLR4 in RAW264.7 cells and the presence of TLR4 in HEK293-TLR4 cells had no effect on the NF-κB luciferase activity induced by rMtb9.8. Thus, we confirmed that the absence of TLR2 affects NF-κB initiation by rMtb9.8 stimulation. The current findings are consistent with observations that rTB10.4, a member of the Mtb9.8–TB10.4 complex^25^, completely abrogated the production of TNF-α in RAW264.7 cells treated with inhibitors of TLR2 or of the NF-κB^16^.

Together, these results demonstrate the role of rMtb9.8 derived from *M. bovis* in regulating immune responses. We have shown that rMtb9.8 activates the NF-κB pathway in RAW264.7 cells through TLR2 and in particular that it induces the nuclear translocation of NF-κB p65 by phosphorylation at Ser536, leading to the downstream production of the inflammatory cytokines TNF-α and IL-1β. Knowledge arising from such investigations should provide a better understanding of the early inflammatory responses that occur during *M. tuberculosis* infection.

### Availability of data and materials statement

Data and materials described in the manuscript are freely available to any scientist wishing to use them.

## Electronic supplementary material

Supplementary Information
