# Supplementary material for: Recombinant Mtb9.8 of Mycobacterium bovis stimulates TNF-α and IL-1β secretion by RAW264.7 macrophages through activation of NF-κB pathway via TLR2
Source: Sci Rep. 2018 Jan 31;8:1928. doi: 10.1038/s41598-018-20433-x (PMC5792469; doi:10.1038/s41598-018-20433-x)

# Recombinant Mtb9.8 of *Mycobacterium bovis* stimulates TNF-α and IL-1β secretion by RAW264.7 macrophages through activation of NF-κB pathway via TLR2

**Shuqing Liu^1,2^, Hong Jia^2^, Shaohua Hou^2^, Ting Xin^2^, Xiaoyu Guo^2^,** **Gaimei Zhang^2^, Xintao Gao^2^, Ming Li^2^, Wuyang Zhu^1*^, Hongfei Zhu^2*^**

**Supplementary Figure 1.** rTB9.8 induces the nuclear translocation of IRF-1. RAW264.7 cells were cultured in the presence of rTB9.8 (5 µg/ml) for the indicated times, and cell lysates of total and nuclear proteins were prepared. Western blot analysis was used to examine the nuclear translocation of IRF-1. TBP and β-actin expression were as control. (A) The full-length blot of IRF-1 expression in the cell lysates of nuclear protein. (B)The full-length blot of TBP expression in the cell lysates of nuclear protein. (C) The full-length blot of β-actin expression in the cell lysates of total protein. The shadow bands under the main bands of β-actin expression were residuals of previous GAPDH expression on the polyvinylidene difluoride membrane, which was washed by the blot stripping buffer (Thermo pierce, #21059) before β-actin expression, blocked with 5% nonfat milk , incubated only with rabbit anti-mouse β-actin Ab and then incubated with HRP-conjugated anti-rabbit IgG secondary Ab. The peroxidase-positive bands were detected using SuperSignal West Dura Extended Duration Substrate and visualized by measuring their chemical luminescence.

(A) The full-length blot of IRF-1 expression


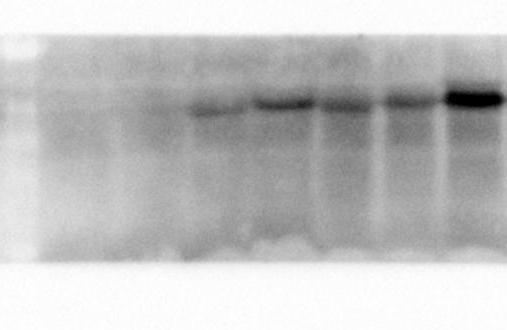


(B) The full-length blot of TBP expression


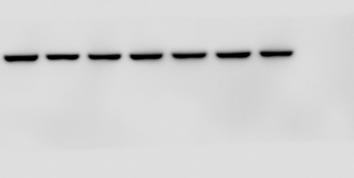


(B) The full-length blot of β-actin expression


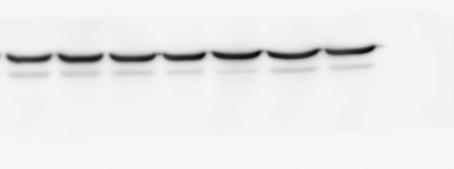

Supplement: Supplementary file 1 — Supplementary Information [file 41598_2018_20433_MOESM1_ESM.doc]
